# Development of a Blended Learning Approach to Delivering HIV-Assisted Contact Tracing in Malawi: Applied Theory and Formative Research

**DOI:** 10.2196/32899

**Published:** 2022-04-19

**Authors:** Nora Ellen Rosenberg, Tapiwa A Tembo, Katherine R Simon, Katie Mollan, Sarah E Rutstein, Victor Mwapasa, Steven Masiano, Hanna E Huffstetler, Vivian Go, Maria H Kim

**Affiliations:** 1 University of North Carolina at Chapel Hill Chapel Hill, NC United States; 2 Baylor College of Medicine Children's Foundation Malawi Lilongwe Malawi; 3 Baylor College of Medicine International Pediatric AIDS Initiative Houston, TX United States; 4 Malawi College of Medicine Blantyre Malawi

**Keywords:** HIV, e-learning, digitial learning, blended learning, digital, contact tracing, assisted partner services

## Abstract

**Background:**

Despite progress toward the Joint United Nations Programme on HIV/AIDS “95-95-95” targets (95% of HIV-positive persons tested, 95% of tested persons on treatment, and 95% of treated persons virally suppressed), a gap remains in achieving the first 95% target. Assisted contact tracing (ACT), in which health workers support HIV-positive index clients to recruit their contacts (sexual partners and children) for HIV testing, efficiently identifies HIV-positive persons in need of treatment. Although many countries, including Malawi, began implementing ACT, testing outcomes in routine settings have been worse than those in trial settings.

**Objective:**

The aim of this paper is to use formative research and frameworks to develop and digitize an implementation package to bridge the gap between ACT research and practice.

**Methods:**

Semistructured qualitative research was conducted in 2019 in Malawi with key informants. Barriers and facilitators to intervention delivery were identified using the Consolidated Framework for Implementation Research. Approaches to digitization were examined using human-centered design principles.

**Results:**

Limited clinic coordination and health worker capacity to address the complexities of ACT were identified as barriers. Ongoing individual training consisting of learning, observing, practicing, and receiving feedback, as well as group problem-solving were identified as facilitators. Important features of digitization included (1) culturally relevant visual content, (2) capability of offline use, and (3) simple designs and basic editing to keep costs low.

**Conclusions:**

Formative research and frameworks played a key role in designing and digitizing an implementation package for ACT delivery in a low-income setting such as Malawi.

## Introduction

In 2014, the Joint United Nations Programme on HIV/AIDS set ambitious “95-95-95” global targets for 2030 [1]. These targets aim to have 95% of persons living with HIV aware of their HIV status, 95% of persons living with HIV on treatment, and 95% of treated persons virally suppressed. Although 81% of persons living with HIV in sub-Saharan Africa (SSA) are now aware of their HIV status, 4.89 million remain undiagnosed, and half a million HIV-exposed infants do not receive timely early infant diagnosis each year [2-5]. Furthermore, a large share of HIV-negative adults remains unaware of being in a relationship with a person living with HIV and are therefore less likely to use effective HIV-prevention strategies [6]. Index-based approaches, in which health workers support persons living with HIV “index clients” to recruit their “contacts” (sexual partners and children) for HIV testing, efficiently identify other persons living with HIV in need of HIV treatment and HIV-negative persons in need of HIV prevention [7]. Index-based approaches build on a fundamental tenet of infectious disease epidemiology: each HIV-positive index has one HIV-positive contact who transmitted HIV to them and other potential contacts who could acquire HIV from them. Index-based approaches have higher diagnostic yields than any other testing approach [7] and hold great promise for achieving the global target of 95% of persons living with HIV tested for HIV [8]. Nonetheless, index-based approaches have not been an integral part of HIV epidemic control in SSA until recently [9].

In 2016, the World Health Organization (WHO) revised its recommendations surrounding index testing from “passive” approaches, in which indexes recruit their own contacts, to voluntary “assisted” approaches, in which health workers support indexes with contact recruitment [10]. These assisted approaches are referred to as “assisted partner notification,” when focused on sexual partners, or “assisted contact tracing (ACT),” when biological children and other household contacts are included. The WHO guidelines provide a “strong recommendation” that assisted contact tracing “should be offered as part of a comprehensive package of testing and care to people living with HIV” [10]. In the years since the WHO guidelines were issued, the President’s Emergency Plan for AIDS Relief (PEPFAR) has promoted index-based approaches that include ACT options [11]. Dozens of countries, including Malawi, have adopted ACT policies and intensified ACT implementation [11,12]. However, real-world ACT testing outcomes have been inferior to those observed in ACT trials [12]. Implementation strategies, guided by theory and formative research, may support translation of ACT outcomes observed from trials into real-world settings.

In this paper, we seek to describe the process of developing an ACT implementation package. We explain the two phases that led its development, which are (1) design and (2) digitization. As qualitative formative research, we did not set out to test hypotheses, but rather to generate them.

## Methods

### Setting

This work was conducted in Malawi, a country in Southeastern Africa with 19 million people. Malawi has a 10.6% adult HIV prevalence and 1.1 million people living with HIV [13]. Malawi has a mature HIV program that is approaching the 95-95-95 targets [13]. Malawi has long had a passive index-based program, with the Department of HIV/AIDS promoting routine distribution of “family referral slips.” In early 2018, through a PEPFAR-led demonstration project, implementing partners began introducing ACT in dozens of facilities, and in early 2019, voluntary assisted partner notification, Malawi’s ACT approach, was adopted as part of Malawi’s national HIV testing policy.

Malawi has a dire human resource shortage with less than 2 physicians, 0.02 psychiatrists, and 0.01 psychologists per 100,000 people, some of the lowest global rates [14,15]. To address its formidable HIV burden with limited human resources, Malawi has task shifted many HIV-related activities, especially counseling tasks, to lay cadres. In 2015, to respond to gaps in HIV testing, Malawi introduced a cadre of HIV diagnostic assistants to improve coverage of early infant diagnosis, viral load testing, and rapid HIV antibody testing [16]. HIV diagnostic assistants are lay persons with secondary education and 4 weeks of preservice training. In its first year, nearly 1200 HIV diagnostic assistants were deployed to 450 facilities, resulting in improved diagnostic indicators [16]. Community health workers hired by local nongovernmental organizations are another lay cadre responsible for a range of HIV-related tasks, including community tracing. HIV diagnostic assistants and community health workers conduct most ACT implementation [17]. HIV diagnostic assistants typically diagnose HIV-positive indexes and support contact elicitation and selection of ACT options, and community health workers typically conduct tracing.

Throughout Malawi’s national program, nongovernmental organizations implementing partners play a critical role in hiring and supervising these lay cadres. Tingathe Program, one of the largest PEPFAR-implementing partners in Malawi, and the implementation lead on this research, was initiated by Baylor College of Medicine Children’s Foundation-Malawi in partnership with the Malawi Ministry of Health. Tingathe takes a family-focused approach to the HIV epidemic by supporting the provision of high-quality, comprehensive HIV services. Tingathe has over 10 years of experience implementing HIV testing, care, and treatment programs in Malawi and currently provides support to Malawi’s HIV care and treatment program with more than 1000 staff in 95 Malawi Ministry of Health facilities.

### Research Procedures

As Malawi’s National HIV testing program evolved from a client-led passive referral model to a provider-supported ACT model, we sought to understand the barriers and facilitators to ACT implementation. This process was guided by the Consolidated Framework for Implementation Research (CFIR) [18]. The CFIR is a determinants framework comprised of five domains (intervention characteristics, individual characteristics, outer and inner organizational settings, and processes) and 39 constructs within these domains that interact with one another [18].

We were also interested in digitizing some or all of these implementation strategies. To facilitate meaningful and sustainable digital programs, technological, human, and cost features must be taken into consideration. We used human-centered design thinking to inform potential adaptations of our implementation strategies into a digital platform. Human-centered design thinking is an approach that integrates the possibilities of (1) technology, (2) the preferences of people, and (3) the requirements for business viability [19] in the process of product develpoment.

In October 2019, we conducted a series of in-person focus group discussions, each 2 hours long, on (1) barriers and facilitators to ACT implementation and (2) the potential role of digital modalities to enhance the program. Tingathe staff were considered key informants and were purposively selected if they held ACT training as well as supervisory and clinical mentorship roles within the organization (N=10). Thus, they had routine contact with hundreds of ACT implementers at dozens of facilities. We also conducted one 2-hour-long, semistructured in-depth interview with a key informant who was not available for the focus group discussions.

All participants provided informed consent to participate. The discussion was facilitated using a semistructured guide. Data were audiorecorded, summarized, and transcribed. Data were reduced into themes organized within the constructs of the CFIR and human-centered design.

### Ethics Approval

The study was reviewed and approved by the National Health Science Research Committee (20/01/2467), the local ethics committee in Malawi, and the Baylor College of Medicine Institutional Review Board (H-47655). Informed consent was obtained from all participants.

## Results

### Population Characteristics

Of the 11 key informants, 3 (27%) were district-level supervisors, 4 (36%) were facility-level supervisors, 2 (18%) were health care workers who provided ACT services, and 2 (18%) were data collection supervisors; 5 (45%) were female, and 6 (55%) were male. All were employed by Tingathe program.

### Themes Surrounding Implementation Package Design

Themes arose within the five following domains of CFIR, which guided our implementation package development process: (1) intervention characteristics, (2) individual characteristics, (3) outer organizational settings, (4) inner organizational settings, and (5) processes.

With respect to intervention characteristics, complexity of ACT implementation was identified as a formidable barrier. Complexities included sensitivities around discussing sexual behavior with indexes, multiple potential contacts for each index, multiple tracing options for each contact, challenges with obtaining correct locator information for sexual contacts, and concerns around index safety.

This level of complexity, along with the minimal preservice training of lay health workers delivering ACT [16], exposed a set of individual characteristics, which were low competence around basic training principles and poor self-efficacy among both health workers and supervisors. For example, Tingathe staff reported that health workers frequently were uncomfortable taking sexual histories, did not understand which partners needed to be tested, and did not know how to help indexes decide which tracing methods to select. Supervisors suggested that modeling these counseling behaviors would enhance skills.

In the outer setting**,** diverse patient needs of both indexes and contacts were not being met, as counseling was conducted generically in a non–client-centered manner and without sensitivity to potential stigma and intimate partner violence or abandonment.

Networks and communication were identified as challenges in the inner setting: health workers in many sections of a single health facility (eg, antenatal care, pediatrics, and antiretroviral therapy) interacted with potential indexes and contacts, but responsibilities were not clearly delineated, and coordination between health workers was minimal.

To address these challenges, several processes were proposed: training that consisted of engaging strategies (role modeling) and planning strategies (practice and feedback) to address perceived intervention complexity, low health worker competence and self-efficacy, as well as patient needs. To address network and communication challenges, group problem-solving approaches that incorporated reflection and evaluation were suggested.

### Resulting Implementation Strategies

**Figure 1:**
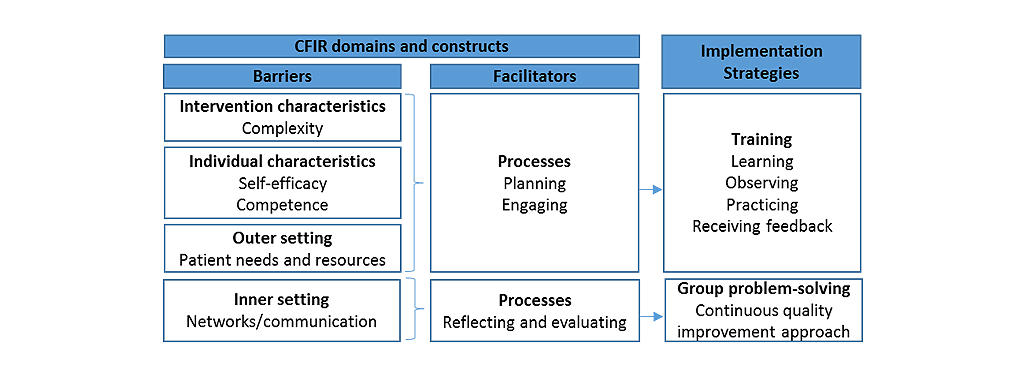
Application of Consolidated Framework for Implementation Research (CFIR).

Synthesizing these findings, we arrived at two sets of implementation strategies, which are as follows: (1) enhanced health worker training to improve ACT counseling sensitivity and competence; and (2) group problem-solving to facilitate ACT coordination (Figure 1). This set of strategies aligns with a seminal review showing the combination of training and group problem-solving to frequently improve health worker practices in low- and middle-income countries (LMIC) [20]. Our specific training and problem-solving approaches were guided by theory and evidence, which will be explained here in detail.

#### Training

Training is the most common implementation strategy in LMICs; however, it is often conducted with suboptimal pedagogical practices and without any ongoing reinforcement [20]. We developed a training guided by the theory of expertise [21], an educational theory that considers deliberate practice and a core set of activities (learning, observing, practicing, and receiving feedback) as important for mastery of new skills [22,23]. Applying this theory to ACT, we developed a set of training activities consisting of the following: (1) explaining ACT counseling skills (learning); (2) modeling ACT counseling skills through vignettes (observing); (3) practicing counseling skills through role plays (practice); and (4) providing suggestions on improvement (feedback). This approach is supported by social cognitive theory, which posits that learning occurs in a social context with reciprocal interactions between the person, environment, and behavior [24]. Observation of modeled ACT vignettes facilitates social learning; practice solidifies behavioral skills and enhances self-efficacy; and feedback refines and reinforces behavioral skills. These activities have enhanced counseling skills across a range of behavioral interventions, including those related to HIV treatment, prevention, and psychosocial support [25-29].

#### Group Problem-solving

Group problem-solving draws on concepts from continuous quality improvement, which is a set of formal and systematic processes to identify and address health systems challenges [30,31]. The purpose is to identify challenges with ACT implementation and coordination, evaluate potential solutions, provide actionable recommendations, and revisit progress through a series of meetings. Similar processes have led to improvements in a range of implementation outcomes in several SSA contexts, including with lay cadres [32-37].

These sets of implementation strategies were combined into an “implementation package” with face-to-face health worker training and group problem-solving. In a previously published work, Tingathe delivered this package to nearly 500 health workers in 36 facilities in Mangochi, Malawi, and observed improvements in a range of ACT indicators: the number of sexual contacts elicited, the number tested, and the number newly identified as HIV-positive [17]. This work demonstrated the value of theory-driven training and problem-solving for improving ACT outcomes.

### Themes Surrounding Implementation Package Digitization

**Figure 2:**
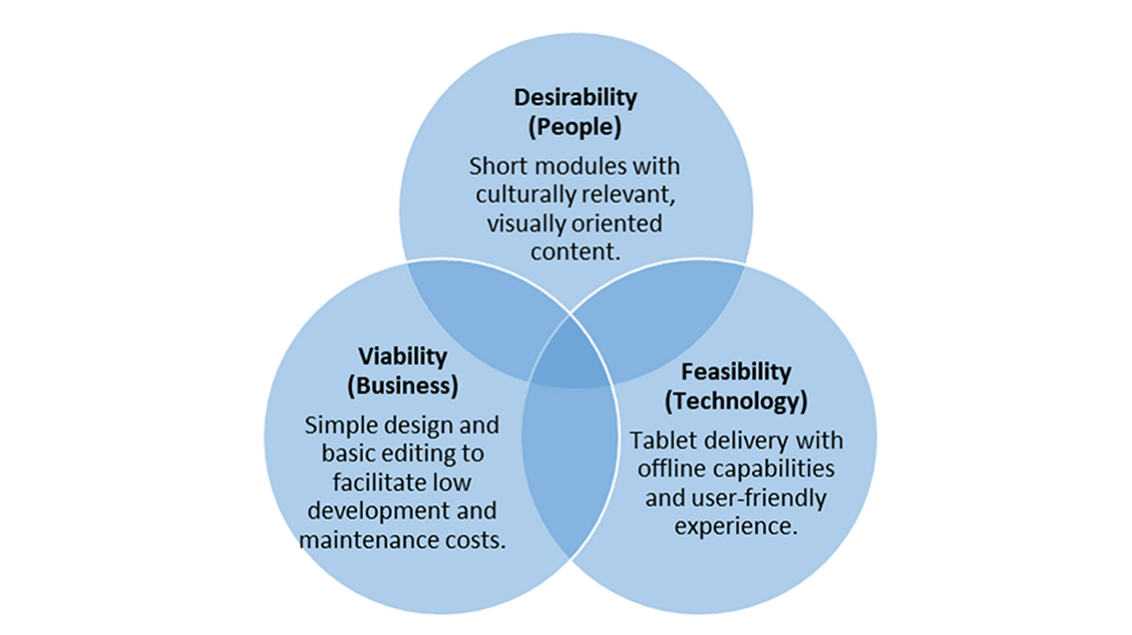
Human-centered design framework.

Themes were also categorized within human-centered design thinking domains, which guided the adaptation of these implementation strategies into a blended learning delivery platform (Figure 2). The results were organized around considerations of (1) technology, (2) desirability, and (3) the requirements for business viability [19].

Applied to rural Malawian health facilities, important technological challenges were discussed, including limited internet connectivity, limited computer literacy, and nonuniversal personal mobile devices, a theme observed in comparable studies in the region [38,39]. These considerations led us to deliberate on content that does not require continuous internet connectivity. They also suggested the importance of user-friendly applications, as has been observed in similar settings [40].

To address desirability for Malawian health workers, participants proposed content that was primarily audio- and video-based to ensure limited reading for this low literacy audience. Studies in similar low-resource environments also proposed similar multimedia elements to address challenges with computer literacy [41]. Participants suggested content be created with Malawian cultural and linguistic considerations in mind. Smaller segments were proposed to enable learning to occur in brief increments when workload permits.

With respect to business aspects, we selected tablets, which are less costly than laptops. We opted for simple visuals and basic editing to limit the budget. For digital learning packages to be cost-effective, it is important to minimize up front development costs, maximize the number of users, or, preferably, do both simultaneously.

### Theme Synthesis: Final Implementation Package

We synthesized findings from the formative work to digitize the package. Based on the WHO’s recommendation that digital modalities for training health workers in LMICs complement, rather than replace, face-to-face learning, we opted for a blended learning approach [42,43]. “Blended learning” combines the best features of digital learning (ie, quality, consistency, and convenience) with the best features of face-to-face learning (ie, interactivity and group engagement) [44,45]. Blended learning is typically more effective than either electronic or face-to-face learning alone for acquiring new knowledge and skills [25,46], and specifically for improving health worker counseling and communication [47-49].

The resulting blended learning implementation package translated our training plus group problem-solving approaches for the possibility of decentralized delivery. We included both individual asynchronous learning sessions and synchronous interactive small-group sessions. The individual learning sessions contain descriptions of ACT skills, video vignettes modeling these skills, and embedded questions assessing comprehension. The small-group learning session contains tablet-guided practice role plays, individual feedback, and facilitated group discussions. It was designed for facility-level conduct guided by ACT data. We developed a model where all health workers involved in ACT would meet to examine their facility’s performance, identify gaps in performance, and propose actionable solutions. Each meeting would review progress toward proposed solutions, as well as their impact on ACT indicators.

## Discussion

In this research trajectory, we used formative research, theory, and frameworks to guide the development of a novel implementation package. First, the consolidated framework for implementation research identified key barriers and facilitators to intervention delivery. Next, the theory of expertise, social cognitive theory, and principles of continuous quality improvement informed the development of an implementation package to address these barriers and facilitators. Finally, human-centered design principles guided the translation of the implementation package from in-person to digital delivery.

The facilitators we identified using the CFIR closely mirrored findings from successful ACT programs in SSA [50]. In 2017-2018, contacts form Kenya and Mozambique accounted for 51% of the 1.7 million contacts tested across 18 SSA PEPFAR countries, even though these countries only accounted for 14% of the population [12]. Similarly, several Cameroonian districts scaled and sustained ACT before the WHO guidelines were published [51]. In an analysis of ACT implementation in these three countries, the intensive ongoing nature of health worker capacity-building was essential [50]. Although the implementation contexts differed, the capacity-building processes were similar. Initial face-to-face trainings imparted ACT counseling skills through skill-based learning and practice role-plays. Ongoing on-the-job mentorship and refresher trainings reinforced and enhanced these skills, consistent with capacity-building best practices in the region [52,53].

Our contributions to ACT implementation science are novel, timely, and noteworthy. In the few years since the WHO issued its guidelines surrounding ACT, there has been widespread ACT implementation in SSA [11,12] and numerous descriptions of barriers, facilitators, and corresponding ACT indicators [50,51,54]. However, the role of theory and frameworks has been limited. Our research trajectory shows the key role that such approaches can play in the design of implementation strategies.

The final blended learning product was pilot tested with promising preliminary results regarding health worker fidelity and preliminary ACT effectiveness [55]. Our next step is to evaluate these ACT implementation strategies in a pragmatic cluster randomized trial to examine both implementation and effectiveness outcomes in a range of clinical settings. This design strength will allow for clear inferences about the impact of the blended learning ACT package on health worker behaviors, clinical indicators, and patient outcomes. This study will also contribute more broadly to the field of HIV implementation science, as only 14% of HIV studies involve implementation research and only 6% are cluster randomized [56].

Our work also represents an important innovation in health worker capacity-building in LMIC settings. A large-scale review of health worker practices in LMIC settings identified the combination of (1) health worker training and (2) group problem-solving as one of the few sets of strategies that routinely improves health worker practices across a range of countries and health conditions [20]. However, this promising combination of strategies has not been translated to a blended learning delivery modality and rigorously evaluated for patient outcomes in LMIC setting. Our research makes this innovative contribution.

Finally, to our knowledge, our planned research trajectory will be the first complete, rigorous evaluation of a blended learning package in a LMIC setting. Although there has been a proliferation of digital and blended learning tools for LMICs over the last decade, most evaluations have focused on proximal outcomes, such as adoption, health worker knowledge, and skills [46]. The WHO has highlighted the critical need for research assessing the impact of blended learning on health worker practices, patient outcomes, and cost-effectiveness in LMIC settings [43]. Our next step is to directly address this set of research questions in a cluster randomized controlled trial. If effective, this implementation strategy will provide an evidence-based solution for adaptation in settings with different characteristics and disease profiles. This research trajectory also represents an important contribution to digital and blended learning.

In recent years, digital learning has proliferated in the health sector, including in LMIC contexts, due to many enticing features, such as the following: (1) learning is not time- or place-dependent; (2) learning does not require an on-site instructor; (3) pace and degree of difficulty can be tailored to the learner; (4) progress and aptitude can be easily monitored; (5) high quality content can be delivered consistently; and (6) infrastructure needs are minimal [44-46]. For in-service training in LMIC contexts, these features are appealing. Digital learning can be delivered at the health facility, eliminating travel and lodging expenses associated with centralized face-to-face trainings. Individual sessions can be delivered asynchronously so all staff are not absent from the clinic simultaneously, minimizing understaffing. New staff can acquire necessary skills right away, rather than waiting for a scheduled training. Health workers can receive high quality instruction without relying on an on-site trainer. Finally, they can potentially be more easily adapted to new settings. Together, these features made digitizing our strategies enticing. Of note, this work was conceptualized in 2019, prior to the SARS-CoV-2 pandemic, but such digital approaches are also appealing for promoting physical distancing and minimizing SARS-CoV-2 exposures [57,58].

Our research has several important limitations. First, this formative study was small. Second, we focused on Tingathe staff (the target population for these implementation strategies), and not on other stakeholders, such as indexes, their contacts, and policy makers. Finally, it was conducted in a single country, and may not be generalizable to others, though these processes can be replicated elsewhere. Adaptation to new settings is an important potential future direction.

In conclusion, our research trajectory illustrates that theories, frameworks, and formative research can play an important role in designing and digitizing implementation strategies. The resulting implementation package provides a promising approach for enhancing ACT and ultimately achieving the 95-95-95 targets in Malawi and beyond.

## Abbreviations

ACT: assisted contact tracing
CFIR: Consolidated Framework for Implementation Research
LMIC: low- and middle-income countries
PEPFAR: President’s Emergency Plan for AIDS Relief
SSA: sub-Saharan Africa
WHO: World Health Organization
